# Pharmacological treatment of early psychosis: risks and benefits

**DOI:** 10.1192/j.eurpsy.2023.121

**Published:** 2023-07-19

**Authors:** L. Sīle

**Affiliations:** ^1^Research and education department, Rigas Centre of Psychiatry and Addiction medicine; ^2^Psychiatry and Addiction medicine, Rigas Stardins University, Riga, Latvia

## Abstract

**Abstract:**

Psychiatry has changed a lot during the last decades and lot of effort has been made to ensure that treatment of schizophrenia spectrum disorders is in line with modern science of medicine. Therefore, in everyday practice the psychiatrist is in a constant process of decision making when to start pharmacological treatment of early psychosis and with what dosage. The lecture will include evidence-based treatment options, the aspects of clinical practice and the power of shared decision-making in psychiatry.

**Disclosure of Interest:**

None Declared

